# Male germline stem cells in non-human primates

**DOI:** 10.5194/pb-4-173-2017

**Published:** 2017-09-22

**Authors:** Swati Sharma, Joana M. D. Portela, Daniel Langenstroth-Röwer, Joachim Wistuba, Nina Neuhaus, Stefan Schlatt

**Affiliations:** 1 Center of Reproductive Medicine and Andrology, Institute of Reproductive and Regenerative Medicine, Albert Schweitzer Campus 1, Building D11, Münster, Germany; 2 Center for Reproductive Medicine, Academic Medical Center, University of Amsterdam, 1105 AZ Amsterdam, the Netherlands; * These authors contributed equally to this work

## Abstract

Over the past few decades, several studies have attempted to decipher the biology of mammalian germline stem cells (GSCs). These studies provide evidence that regulatory mechanisms for germ cell specification and migration are evolutionarily conserved across species. The characteristics and functions of primate GSCs are highly distinct from rodent species; therefore the findings from rodent models cannot be extrapolated to primates. Due to limited availability of human embryonic and testicular samples for research purposes, two non-human primate models (marmoset and macaque monkeys) are extensively employed to understand human germline development and differentiation. This review provides a broader introduction to the in vivo and in vitro germline stem cell terminology from primordial to differentiating germ cells. Primordial germ cells (PGCs) are the most immature germ cells colonizing the gonad prior to sex differentiation into testes or ovaries. PGC specification and migratory patterns among different primate species are compared in the review. It also reports the distinctions and similarities in expression patterns of pluripotency markers (OCT4A, NANOG, SALL4 and LIN28) during embryonic developmental stages, among marmosets, macaques and humans. This review presents a comparative summary with immunohistochemical and molecular evidence of germ cell marker expression patterns during postnatal developmental stages, among humans and non-human primates. Furthermore, it reports findings from the recent literature investigating the plasticity behavior of germ cells and stem cells in other organs of humans and monkeys. The use of non-human primate models would enable bridging the knowledge gap in primate GSC research and understanding the mechanisms involved in germline development. Reported similarities in regulatory mechanisms and germ cell expression profile in primates demonstrate the preclinical significance of monkey models for development of human fertility preservation strategies.

## 1 Introduction

In adult men, spermatogonial stem cells (SSCs) are the foundation of fertility since they are able to drive spermatogenesis by self-renewal and differentiation throughout adulthood. Consequently, the loss or damage of SSCs or their developmental progenitors leads to an impaired spermatogenic function, as observed in prepubertal cancer survivors after gonadotoxic treatments or those suffering from genetic causes like Klinefelter's syndrome.

Over the past few years several experimental approaches have been explored to preserve and restore fertility of prepubertal boys following gonadotoxic treatments. Among these are (1) autologous transfer of germ cell suspensions into seminiferous tubules, (2) in vitro differentiation of germ cells in cell or organ culture systems, (3) autologous grafting of testicular tissue and (4) xenografting of testicular tissue into nude mice (for reviews see Schlatt et al., 2009; Stukenborg et al., 2014; Wyns et al., 2010). Most of these methods were used successfully for generation of rodent sperm, but they could not be successfully employed for derivation of human spermatozoa (Brinster and Zimmermann, 1994; Stukenborg et al., 2008, 2009; Sato et al., 2011, 2013; Yokonishi et al., 2013). Therefore more preclinical research is required to establish these experimental approaches for fertility preservation before these can be adapted in clinical settings.

Primordial germ cells (PGCs) are defined as embryonic precursors of male and female gametes. In males, once these cells are located within seminiferous tubules, they are termed gonocytes. Following migration of these cells to the basal membrane of the seminiferous tubules they are referred to as prespermatogonia or spermatogonia, depending on whether these cells are in limited or full contact with the basal lamina, respectively. A subpopulation of these spermatogonia will develop into SSCs which have the ability to self-renew and to differentiate into spermatozoa.

In previous publications different terms have been used for stem cell populations which can be found in or isolated from immature or adult testicular tissue. The term SSC has also been used for cultured cells, especially when germ cell transplantations were applied to confirm stem cell characteristics (Sadri-Ardekani et al., 2009, 2011; Nickkholgh et al., 2014; Valli et al., 2014; Hermann et al., 2012). Other publications use a more general, term germline stem cells (GSCs), for diploid germ cells from immature and adult testes which can be expanded in vitro (Conrad et al., 2008; Ko et al., 2006) and which after transplantation can reinitiate spermatogenesis in germ-cell-depleted testes (Kanatsu-Shinohara et al., 2003; Ogawa et al., 2004). In this context the term GSCs designates stem cell populations which have been derived from germline cells. Especially for cells isolated from immature testes, this term is more appropriate, since in situ non-self-renewing divisions of primitive germ cells occur before spermatogenesis is initiated during puberty. Consequently, immature germ cells are by definition not SSCs but rather progenitors of SSCs.

Mouse GSCs have been extensively studied in situ and in vitro on morphological, molecular and functional levels (for review see Komeya and Ogawa, 2015). Briefly, mouse SSCs have been characterized in situ as GFRα1 (GDNF receptor alpha 1) expressing subpopulation of type A spermatogonia (Tegelenbosch et al., 1993; Nakagawa et al., 2010). In addition, protocols for isolation and in vitro propagation of GSCs have been developed (Kanatsu-Shinohara et al., 2003, 2005; Kubota et al., 2004; Ogawa et al., 2004), and it has even been shown that under suitable medium conditions the derivation of pluripotent cells from cultured GSCs is possible (Kanatsu-Shinohara et al., 2004; Ko et al., 2006). The development of mouse GSC cultures was a huge advance for GSC research, since they allowed studying the direct effect of cytokines (Kanatsu-Shinohara et al., 2005) and chemokines (Dovere et al., 2013) on GSCs in vitro, which correspond to the SSC population in situ. Consequently, downstream signaling pathways of cytokines and chemokines and the transcriptional regulation of SSCs could be analyzed for the first time using these in vitro systems (Braydich-Stolle et al., 2007; Lee et al., 2007; Oatley et al., 2007; Ishii et al., 2012). Apart from that, the in vitro reconstruction of SSC niches allowed investigating the effect of specific niche factors (Kanatsu-Shinohara et al., 2012).

In contrast to the wide knowledge which has been collected on mouse GSCs, information about the molecular identity and regulation of human and non-human primate (NHP) GSCs is still limited, and protocols for their isolation and in vitro propagation are still questioned. Due to the existence of different SSC systems in rodents and primates, advances made in mouse GSC research cannot be translated to human and NHP models. The primary distinction is the presence of a progenitor stem cell population in primate species. In rodents SSCs undergo highly synchronous mitotic and meiotic divisions, and A${}_{\mathrm{single}}$ differentiate into A${}_{\mathrm{paired}}$, further dividing into chains of up to 16 cells; whereas in primates, stem cells are classified as A${}_{\mathrm{dark}}$, the irregularly dividing reserve stem cells, and A${}_{\mathrm{pale}}$, the self-renewing population. These cells further undergo mitotic and meiotic divisions to differentiate into spermatozoa (Clermont and Leblond, 1953, 1959; Clermont and Bustos-Obregon, 1968; Huckins, 1971; Oakberg, 1971; Ehmcke et al., 2006; Ehmcke and Schlatt, 2006).

Since the impact of findings in rodent models on humans is limited due to the fundamental differences in the spermatogonial identity and expansion, NHP models represent important and more expressive model organisms in preclinical GSC research. This review summarizes advances which have been made during the last decade in primate germ cell specification, migratory pattern, SSCs, their developmental progenitors and plasticity potential.

## 2 Germ cell specification and migration in primates

In contrast to mouse PGCs, comparatively few studies have investigated the processes of specification and migration of human PGCs to date (De Felici, 2013). In mammals, germ cells are specified during early embryonic development by extracellular signaling (epigenesis) from somatic cells and are referred to as PGCs. Human PGCs can be detected in the caudal wall of the yolk sac 3 weeks following conception (De Felici, 2013). These cells translocate from an extra-embryonic to the intra-embryonic position in the developing gonad. While the interplay of chemokine CXCL12 with its receptor CXCR4 has been shown to be important for PGC migration in mice (Molyneaux et al., 2003), the regulatory processes in primates remain unknown up to now. However, we have recently demonstrated that chemokine CXCL12 and its receptors can also be detected in marmosets and humans throughout testicular development, indicating that the regulation of PGC migration may be evolutionarily conserved (Westernströer et al., 2015).

## 3 Animal models to study early germ cell development in the human

Due to ethical and legal limitations regarding the accessibility of human embryos and testicular tissues, the majority of studies focusing on early testicular development have been performed in rodents. However, as outlined above, species-specific differences especially with regard to the SSC system limit transferability of these findings to humans. For this reason, NHP models (like marmoset, rhesus and cynomolgus monkeys) which share similar testicular developmental patterns and common SSC system are significant for research studies.

The marmoset monkey (*Callithrix jacchus*), for example, presents a more suitable model for studying germ cell development (Li et al., 2005; Mitchell et al., 2008; Albert et al., 2010, 2012; Lin et al., 2012; McKinnell et al., 2013). For instance, a common hallmark of human and marmoset testes is that gonocytes are still present in the testes of a newborn until a couple of weeks after birth (Wistuba et al., 2003; Mitchell et al., 2008). This is in contrast to testes from newborn rhesus and cynomolgus monkeys, which contain type A spermatogonia as the predominant germ cell type (Simorangkir et al., 2005). Whereas rodent testes exhibit mostly gonocytes at the time of birth, these undifferentiated germ cells then quickly differentiate into type A spermatogonia or enter apoptosis (de Rooij and Grootegoed, 1998; Forand et al., 2009; Wu et al., 2009).

Both marmoset and macaque monkeys have been extensively used as a NHP model in a number of studies to evaluate the expression of germ cell marker genes in early postnatal testes. However, it is also important to note that certain physiological differences need to be considered. For instance, gestation in marmoset monkey takes only 143–145 days, while in macaques it ranges between 160 and 175 days. This is in contrast to human gestation, which takes about 267 days (Silk et al., 1993; Aeckerle et al., 2015). Furthermore, despite this comparatively short gestation period, the processes of PGC specification and migration are still significantly delayed in marmoset monkeys (Phillips, 1976; Merker et al., 1988; Li et al., 2005).

## 4 Germ cell expression profile in primates

### 4.1 Comparative expression analysis in primordial and perinatal period

**Figure 1:**
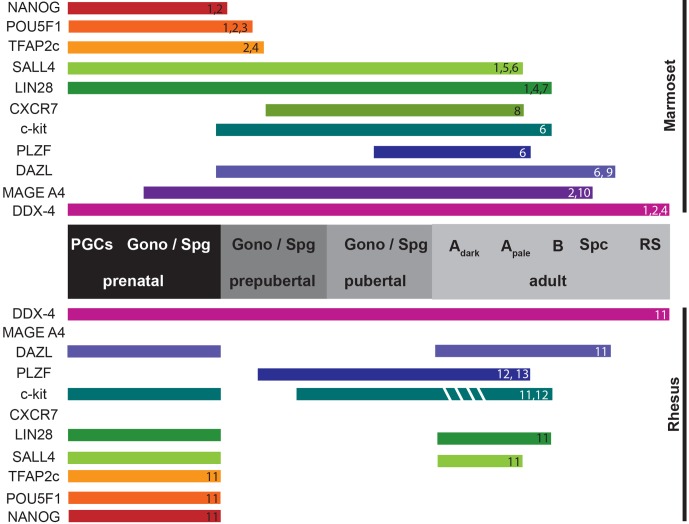
Schematic representation of comparative expression profiles of prenatal, perinatal and adult germ cell markers in marmoset and rhesus monkeys. Prenatally expressed markers constitute primordial germ cells (PGCs), the embryonic precursors of male gametes, and gonocytes (Gono), cells located in seminiferous tubules. Prespermatogonia are the cells migrating to the basal lamina, whereas spermatogonia (Spg) are already in direct contact with basal lamina. A sub-population of these spermatogonial stem cells further mature into more differentiated cells including B spermatogonia (B), spermatocytes (Spc), round (RS) and elongated spermatids, and finally into spermatozoa. Expression patterns of various germ cell markers across different developmental stages are represented by colored bars (1: Aeckerle et al., 2015; 2: Mitchell et al., 2008; 3: McKinnell et al., 2013; 4: Albert et al., 2010; 5: Eildermann et al., 2012a; 6: Lin et al., 2015; 7: Aeckerle et al., 2015; 8: Westernströer et al., 2015; 9: Lin et al., 2012; 10: Eildermann et al., 2012b; 11: Sasaki et al., 2016; 12: Hermann et al., 2007; 13: Hermann et al., 2009).

Employing the common marmoset as a NHP model, Aeckerle et al. (2015) recently performed a systematic study to evaluate the process of PGC migration. As morphological identification of PGCs proved to be difficult due to their heterogeneous morphology, immunohistochemical stainings for pluripotency markers OCT4A (which belongs to POU class 5 homeobox 1 – POU5F1), NANOG (Nanog homeobox), SALL4 (Sal-like protein 4) and LIN28 (Lin-28 homolog A) were performed at embryonic (E) days E50, E65, E72, E75 and E95 in pre-implantation embryos. Especially, OCT4A and NANOG antibodies facilitated the reliable identification of PGCs. Notably, the homogenous DDX4 expression in marmosets, in contrast to humans, facilitated the confirmation of the germ cell origin in marmoset monkey testes (Anderson et al., 2007; Gkountela et al., 2013). In contrast, SALL4 and LIN28 were almost ubiquitously expressed at the early developmental time point of E50. By E65 PGCs were immunopositive for OCT4, NANOG, SALL4 and LIN28 and were located at extra-gonadal sites as well as in the forming gonads. Expression of these four markers was maintained until E95, when all germ cells were located in the gonad and more specifically the majority were located within testicular cords (Aeckerle et al., 2015). The gradual downregulation of pluripotency factors OCT4A and NANOG starts at the time when Sertoli cells enclose the male PGCs (Shamblott et al., 1998). Expression patterns of OCT4A, NANOG, SALL4 and LIN28 during embryonic stages in marmosets have been represented in Fig. 1.

Regarding the migratory process of marmoset PGCs, Aeckerle et al. (2015) found that on day E50 PGCs are located not only in the caudal endoderm lining the yolk sac stalk but also in tissues neighboring the gonadal ridges. Based on this finding, they proposed that the subsequent translocation of PGCs may rather be achieved by growth and morphological changes of these tissues resulting in a passive guidance towards the gonadal anlage (Wrobel and Süss, 1998). Consequently, short-range migration might play a more important role in these species than long-range PGC migration (Aeckerle et al., 2015).

Focusing on marker expression in fetal and early postnatal germ cells, Mitchell et al. (2008) performed a comparative analysis on human and marmoset germ cells. They reported that the testicular tissue of 11-week-old marmosets consisted of cords surrounded by an interstitial compartment, which resembled human fetal testis histology (Gaskell et al., 2004; Mitchell et al., 2008). Expression of pluripotency markers TFAP2C (Transcription Factor AP-2 Gamma), OCT4 and NANOG was detected in a large number of germ cells at this early developmental stage. During subsequent development in marmosets, the number of immunopositive cells decreased, resulting in a small proportion of immunopositive germ cells at the time of birth. Postnatally, the expression of NANOG persisted in marmosets until the neonatal period, whereas individual OCT4+ and TFAP2C+ cells were observed until 6 weeks after birth (Mitchell et al., 2008). These findings are in agreement with a previous report indicating that downregulation of NANOG precedes that of OCT4 (Hoei-Hansen et al., 2005). Histological evaluation of neonatal human testes revealed comparable OCT4 expression pattern (Mitchell et al., 2008; Reijpert-de Meyts et al., 2004). The mechanisms responsible for the downregulation of OCT4 have not been unraveled so far. Prior to migration of cells into the stem cell niche at the basement membrane of seminiferous tubules, some critical steps (OCT4 downregulation and central localization of OCT4+ cells in the seminiferous cords) might prevent the pluripotent identity of cells, thereby avoiding development into in situ carcinoma (Honecker et al., 2004; Cools et al., 2005; McKinnell et al., 2013). Interestingly, the expression of MAGE A4 (melanoma antigen family A4) and DDX4 (markers for more differentiated germ cells) revealed a complementary expression pattern compared to the pluripotency markers. Immunopositive cells could not be detected until 15 weeks in marmosets, and even then expression was restricted to a small subpopulation of cells. However, the proportion of positive cells continuously increased so that the majority of cells showed expression of these two markers at the time of birth (Mitchell et al., 2008). Again, the marker expression pattern was similar in humans apart from the fact that the temporal development is not identical. In humans, DDX4+ cells cannot be detected until the second trimester and are not observed before colonization of gonadal ridges (Anderson et al., 2007; Mitchell et al., 2008; Gkountela et al., 2013). PGCs observed during the third week of differentiation show positive expression for DDX4 and c-kit, which is also detected on the surface of these cells. OCT4A localization is observed in the nucleus of c-kit+ cells at 7 to 10.5 weeks. Exclusive localization is observed for c-kit and DDX4 from 12.5 weeks onwards with only 10 % of the cell population showing co-localization for both markers. During the second trimester a gradual transition of the c-kit and OCT4 expression pattern is observed, which changes from cytoplasmic to nuclear co-localization (Gkountela et al., 2013).

In fetal and neonatal marmoset monkey testes, co-localization experiments for undifferentiated and differentiated germ cell markers revealed that their expression was synchronized neither within nor between the seminiferous cords. Rather, the presence of distinct subpopulations including OCT4+/DDX4-, OCT4+/DDX4+ and OCT4-/DDX4+ cells was detected until a few weeks after birth (Gaskell et al., 2004; Mitchell et al., 2008; McKinnell et al., 2009). This finding is in agreement with data from other studies focusing on human germ cell development (Honecker et al., 2004; Anderson et al., 2007; Gaskell et al., 2004; Pauls et al., 2006). In macaques, the premigratory cell subpopulation consists of DDX4-/OCT4+ cells, whereas the DDX4-/OCT4+ and DDX4+/OCT4- subpopulation appears in early and late post-migratory stages as represented in Fig. 1 (Sasaki et al., 2016).

Another publication by Albert et al. (2010) started a debate regarding the expression pattern of OCT4 in marmoset testes between birth and 8 weeks of age. Whereas the proportion of OCT4+ cells was decreased in 8-week-old testes, the absolute number of these immature cells per testis remained constant. In contrast, the number of DDX4+ cells was significantly higher (about twofold). Albert et al. (2010) proposed that the perceived loss of OCT4+ cells was the result of the threefold extension of seminiferous cords during this stage, resulting in fewer OCT4+ cells per cross section (Albert et al., 2010). Additionally, co-stainings for OCT4, TFAP2C and DDX4 revealed the presence of five different subpopulations of undifferentiated germ cells (Albert et al., 2010).

The subject of perinatal germ cell development in marmosets, humans and rats was revisited by McKinnell et al. (2013) in particular with regard to the expression of OCT4. Performing a systematic evaluation of fetal and postnatal marmoset testicular tissues obtained at an age of 0.5 to 22 weeks, this study focused on the quantification of germ cells expressing markers including OCT4 and DDX4 and determination of the proliferation index. It was reported that germ cell numbers increased 2.7-fold (between 0.5 and 2.5 weeks of age) due to the increasing numbers of DDX4+ rather than the OCT4+ germ cells, which decreased. During subsequent testicular development (from 2.5 and 5–7 weeks of age), germ cell numbers did not increase, yet a further decline of OCT4+ cells was observed, and in four out of nine animals OCT4 immuno-expression was no longer detectable (McKinnell et al., 2013). Surprisingly, though, evaluating the proliferation index of OCT4+ and DDX4+ cells revealed that in fetal testes and in the first weeks after birth the proliferation index of these two subpopulations was similar, which is in contrast to the declining number of OCT4+ cells. One proposed explanation is that OCT4 expression is downregulated during the proliferation process or that these OCT4+ cells increasingly enter apoptosis (McKinnell et al., 2013). These findings contradict the results published by Albert et al. (2010), indicating that the use of an unspecific OCT4 antibody may have led to these conflicting results (Warthemann et al., 2012). The size of the population of OCT4+ cells appears to continuously decrease from late fetal life until 2.5 weeks after birth and completely disappear by the end of mini-puberty (McKinnell et al., 2001, 2013). Current data therefore suggest an ongoing process of germ cell differentiation in neonatal testes, rather than the maintenance of a stable OCT4 population.

### 4.2 Comparative expression analyses of spermatogonial markers

As outlined above, the population of undifferentiated spermatogonia consists of A${}_{\mathrm{dark}}$ and A${}_{\mathrm{pale}}$ spermatogonia. In rodents, germ cell transplantation assay presents a routine method to unambiguously demonstrate the SSC identity (Ogawa et al., 1997; Nagano and Brinster, 1998). However, as primate-to-primate germ cell transplantations cannot be routinely used to evaluate the presence of primate spermatogonia and as primate-to-mouse germ cell transplantations only provide limited information regarding the SSC identity, the identification of unambiguous spermatogonial markers remains a main objective in reproductive biology.

The cytoplasmic RNA-binding protein LIN28 increases translation of mRNAs including pluripotency factor OCT4. Functionally, it plays a role in maintaining the undifferentiated state in human embryonic stem (ES) cells (Qiu et al., 2010; Peng et al., 2011). In mice, Lin28 has been demonstrated to be important for PGC specification (West et al., 2009), and its expression is maintained in gonocytes and in immature and adult spermatogonia throughout testicular development (Zheng et al., 2009; Aeckerle et al., 2012). Seeking to evaluate if this expression pattern is evolutionarily conserved, Aeckerle et al. (2012) compared the expression pattern of LIN28 in mice, marmosets (*Callithrix jacchus*) and macaques (*Macaca mulatta)* at different stages of testicular development, which is represented in Fig. 1. In marmosets, strong expression of LIN28 was detected in PGCs (Aeckerle et al., 2015) as well as in gonocytes. This expression was maintained postnatally, although the number of immunopositive cells decreased throughout testicular development. While 100 % of seminiferous tubules contained LIN28+ germ cells in newborn testes, only 90 % and 0.5–2.3 % of tubules contained positive germ cells in pubertal and adult testes, respectively (Aeckerle et al., 2012). Morphological evaluation in adult testes revealed that LIN28 was expressed by subpopulations of A${}_{\mathrm{dark}}$, A${}_{\mathrm{pale}}$ and B spermatogonia, demonstrating that LIN28 is not selectively expressed by these morphologically defined subtypes (Aeckerle et al., 2012). Data regarding the putative functional relevance of this marker expression were obtained by studying monkey testes during seasonal involution. Interestingly, those stem cells that re-initiate spermatogenesis following spermatogenic quiescence expressed LIN28, indicating that this cell population represents the SSC pool (Aeckerle et al., 2012). Inter-species comparison generally revealed an evolutionary conservation. However, while LIN28+ spermatogonia could be detected in several tubular cross sections in adult mouse testes (Aeckerle et al., 2012), only few tubular cross sections contained positive cells in adult marmoset, human and rhesus testes (Aeckerle et al., 2012).

The zinc finger transcription factor SALL4 is another factor involved in the regulation of pluripotency, specifically by regulating OCT4 expression and interaction with NANOG (Zhang et al., 2006; Rao et al., 2010). The finding that loss of SALL4 results in death of mouse embryos prior to implantation (Elling et al., 2006; Sakaki-Yumoto et al., 2006; Tsubooka et al., 2009) demonstrates a crucial role for early embryo development. Based on the observation that SALL4 expression in adult mice was only maintained in reproductive organs (Kohlhase et al., 2002; Tsubooka et al., 2009), Eildermann et al. (2012a) performed a comprehensive study analyzing the expression of SALL4 throughout testicular development in mice, marmosets, humans and rhesus monkeys. In marmosets, nuclear staining was detected in late PGCs, gonocytes and spermatogonia, with different staining intensities between and also within the cell populations. Importantly, some germ cells remained immunonegative (Eildermann et al., 2012a). The proportion of SALL4+ germ cells decreased during puberty and the associated growth of tubular cords: a visual thinning effect that has previously been reported regarding the number of OCT4+ cells (Albert et al., 2010). Finally, in pubertal and adult testes, SALL4 expression was restricted to A${}_{\mathrm{dark}}$ and A${}_{\mathrm{pale}}$ spermatogonia, showing distinct staining intensities within the respective cell populations and in addition to the nuclear staining also low expression in the cytoplasm of some cells (Eildermann et al., 2012a). In the limited immature human testicular samples, SALL4 expression was demonstrated in the majority of gonocytes in fetal human testes and in pre- and type A spermatogonia of a 1-year-old boy. In normal adult human testes a strong SALL4 expression was detected in a subpopulation of spermatogonia. Consequently, the expression pattern in adult mouse, rhesus monkey and human testes are generally in agreement with data obtained from marmoset monkey as demonstrated in Fig. 1 (Eildermann et al., 2012a). Although the functional importance of SALL4 in male germ cells remains to be elucidated, immunohistochemical data show a strong but heterogeneous expression in undifferentiated germ cell types. This might indicate that this marker is rather associated with the developmental potential of spermatogonia instead of the morphological characteristics which define A${}_{\mathrm{dark}}$ and A${}_{\mathrm{pale}}$ subpopulations (Eildermann et al., 2012a).

Immunohistochemical detection of GFRα and PLZF (promyelocytic leukemia zinc finger), which are reportedly expressed in undifferentiated spermatogonia in rodents, revealed that these markers are also expressed in adult rhesus testicular tissues (Meng et al., 2000; Buageaw et al., 2005; Ryu et al., 2005; Buaas et al., 2004; Costoya et al., 2004). Quantification of PLZF immunopositive cells per seminiferous cross section showed that 1.84±0.59 cells were positive, which is slightly less than the combined number of A${}_{\mathrm{dark}}$ and A${}_{\mathrm{pale}}$ spermatogonia per cross section, indicating that this marker is expressed by a subpopulation of undifferentiated spermatogonia in rhesus monkey as shown in Fig. 1 (Hermann et al., 2007, 2009).

Assuming that A${}_{\mathrm{dark}}$ and A${}_{\mathrm{pale}}$ spermatogonia can be distinguished based on their mitotic activity (Ehmcke et al., 2005; Simorangkir et al., 2009), a co-localization study was performed evaluating the expression of spermatogonial markers with the proliferation marker Ki67 (Lin et al., 2015). Two populations of spermatogonia were identified in adult marmoset monkeys with the molecular phenotype of SALL4+/PLZF+/LIN28+/DPPA4+ (developmental pluripotency-associated 4)/DAZL+ (deleted in azoospermia-like) and DAZL+/c-kit+/KI67+ (Lin et al., 2015). While the former population may represent A${}_{\mathrm{dark}}$ spermatogonia, the latter population may comprise the A${}_{\mathrm{pale}}$ population. However, Lin et al. (2015) questioned the possibility to differentiate these two populations based on this one criterion.

Finally, cell surface glycans SSEA4 (anti-stage-specific embryonic antigen 4) and TRA-1-81 were reported to stain spermatogonia, in marmoset and macaque testes (*Macaca silenus* and *Macaca mulatta*). Based on the observation that a smaller population of cells was immunopositive for TRA-1-81 than for SSEA4, it was suggested that the former marker may be more specific for SSCs (Müller et al., 2008). Additionally, expression of TRA-1-60 was detected in marmoset spermatogonia (Müller et al., 2008). It is important to note, though, that none of these markers was detected in human testes. Functional data were provided by another study, comparing the colonization efficiency of sorted SSEA4+ cells to non-sorted and sorted triple-stained cells (CD49f+/CD90+/CD117-) from adult rhesus monkeys. Interestingly, exclusively the SSEA4+ fraction showed a significantly enriched colonization efficiency. Regarding interpretation of these results, it was suggested that the triple-stained cells may consist of a rather quiescent SSC population, whereas SSEA4 may mark actively proliferating SSCs (Maki et al., 2009).

Also, based on rodent studies, expression of markers THY-I (Thymocyte Antigen 1), GFRα, PLZF, NGN3 (Neurogenin 3) and c-kit was assessed in juvenile and adult rhesus macaques. In particular THY-I positive cells from juvenile testes were confirmed to contain GSCs when they were enriched and transplanted into germ-cell-depleted nude mouse testes (Hermann et al., 2009). Co-stainings for THY-I, GFRα, PLZF and NGN3 revealed an incomplete overlap for all marker combinations, indicating the presence of different subpopulations of type A spermatogonia in juvenile and adult testes (Fig. 1). However, none of these markers was restricted to the population of A${}_{\mathrm{dark}}$ or A${}_{\mathrm{pale}}$ spermatogonia, and these morphological phenotypes may be associated with a cell cycle stage as opposed to distinct stem cell functions. Interestingly, data indicate that NGN3 is associated with the transition from a c-kit- to a c-kit+ state (Hermann et al., 2009, Fig. 1). This finding is supported by rodent data showing that NGN3 is expressed from A${}_{\mathrm{aligned}}$ to B spermatogonia, which are also positive for c-kit (Yoshida et al., 2004, 2007; Manova et al., 1990; Schans-Strassen et al., 1999).

In conclusion, expression of individual spermatogonial markers can be detected in the testes of rodents, NHP and humans. All studies report some overlap and a heterogeneous expression pattern within these cell populations. However, the distinctions make it necessary to investigate the expression profile in each species of interest. It has also been suggested that undifferentiated spermatogonia have the potential to either differentiate or acquire stemness potential (Eildermann et al., 2012a).

## 5 Germ cell plasticity

Derived from pluripotent embryonic cells, mammalian germ cell lineage specification occurs in response to secreted factors from neighboring somatic cells. Several signaling pathways are found to be conserved among rodents and primates. PGC specification initiates during post-implantation stage and is induced by BMP (bone morphogenetic proteins) and Wnt signaling from extraembryonic ectoderm and visceral endoderm, respectively (Ginsburg et al., 1990; Lawson et al., 1999; Ohinata et al., 2009). In mice it occurs at 5.5–6 days, while in humans it occurs at 2–3 weeks of embryonic development. Expression of BMP4 and WNT3A was also observed to be significant for monkey PGC specification (Sasaki et al., 2016).

WNT3 activates mesodermal factor T, which is required for mouse PGC specification through regulation of BLIMP1 and PRDM14. BMP signals act through SMAD1 and SMAD5; on dimerization with SMAD4 they induce PGC transcriptional regulators. PGC specification is initiated at embryonic day 6.25 with the expression of BLIMP1, followed by induction of TFAP2C. In combination with PRDM14, BLIMP1 and TFAP2C are the key regulators involved in PGC specification.

To understand molecular mechanisms involved in reprogramming to a pluripotent state, it is necessary to mimic germ cell developmental patterns in vitro. Several groups have demonstrated that cell fate could be reprogrammed using transcription factors (OCT3/4, SOX2, KLF4 and MYC) in mice to induce pluripotency (Takahashi et al., 2007). Mouse induced pluripotent stem cells (iPSCs) can be reprogrammed into PGC-like cells. These in vitro generated cells can be further developed into oocyte-like or haploid male gametes with fertilization competence once an in vivo gonadal environment is provided (Zhou et al., 2016; Ishikura et al., 2016).

Unlike mouse ES cells and iPSCs, which show comparable pluripotent characteristics, it remains difficult to generate primate pluripotent cells due to their heterogeneous cell population and differentiation potential (Takahashi and Yamanaka, 2016). Recent studies report generation of mesoderm-like cells from human iPS cells, which can be induced to produce primordial germ-like cells (Sasaki et al., 2015). In vitro generation of Sertoli-like cells and haploid spermatids has been reported using human umbilical cord perivascular cells, which demonstrates differentiation potential of these cells (Shlush et al., 2017). Although the functional competence of these in vitro generated cell types still remains to be validated, the transcriptome analysis of in vitro generated human PGCs shows comparable expression patterns to cynomolgus monkey PGCs derived from early amnion (Sasaki et al., 2016). Attempts to characterize germ cell populations from monkey ES cells demonstrates similar expression patterns to those observed for in vitro generated human PGCs. It is also speculated that amnion might be giving rise to germ cell lineage in cynomolgus monkey; this could also be the case in humans (Sasaki et al., 2016). In cynomolgus monkeys, ES cells show DDX4 expression during primordial germ cell development. Increased DDX4 expression in cultured embryoid bodies shows that monkey ES cells can acquire germ cell lineage (Yamauchi et al., 2009). Some other studies also provide evidence for spermatogonial plasticity among primates (Ehmcke and Schlatt, 2006).

Recent studies suggest that plasticity behavior of cycling stem cells is observed not only in testicular tissue but also in other organ systems. For instance, the dynamics involved in self-renewal and differentiation of neuronal, hair and intestinal stem cells have been investigated employing live imaging and lineage tracing approaches (Krieger and Simons, 2015). Like in testes, other organs also show the presence of a reserve stem cell population that only divides into active stem cells during injury. Additionally, studies investigating the self-renewal of different mammalian tissues (intestine, hair, skin) clearly indicate a dependence on Wnt signals. This suggests the existence of an intrinsic program and involvement of signaling proteins in regulating stem cell homeostasis (Clevers et al., 2014). Since the discovery of iPSC technology, reprogramming of somatic cells into pluripotent stem cells has been widely used as a tool to understand cellular plasticity.

## 6 Conclusion

Divergent findings in mice and primates may be related to the different SSC systems and not to distinct functions. In primates, developmental potential of spermatogonia is represented by heterogeneous expression of undifferentiated germ cell markers rather than the morphological characteristics of active and reserve stem cell populations (Eildermann et al., 2012a). Distinct stem cell systems among primates and the presence of a more heterogeneous germ cell population are major challenges for their characterization. For instance, to date no marker has been identified that facilitates the distinction of A${}_{\mathrm{dark}}$ and A${}_{\mathrm{pale}}$ spermatogonia. It has also been suggested that undifferentiated spermatogonia may be able to switch from one type to another (Eildermann et al., 2012a). The distinct reaction of individual stem cell populations to external stimuli is a functional hallmark representing phenotypic heterogeneity of stem cells. However, distinct expression patterns in humans and NHP clearly demonstrate that findings from one primate model cannot be directly translated to another.

Compared to information on rodents, information on primate germ cell specification and characteristics is still limited. Humans and NHPs share a common stem cell progenitor system as well as similar germ cell developmental patterns. Therefore, NHPs come across as a suitable model for biological and preclinical research. However, the specific role or function of key regulators in primate PGC specification is still not clear. Recapitulating primate germ cell development in vitro would enable us to understand regulatory pathways involved in male gametogenesis and devise strategies for male infertility treatment.

## Data Availability

No data sets were used in this article.
